# Electrospun Quercetin-Loaded PLA and PLA/Polyethylene Glycol Fibers: Preparation, Characterization, and In Vitro Evaluation

**DOI:** 10.3390/pharmaceutics17050577

**Published:** 2025-04-27

**Authors:** Nikoleta Stoyanova, Nasko Nachev, Ani Georgieva, Reneta Toshkova, Mariya Spasova

**Affiliations:** 1 Laboratory of Bioactive Polymers, Institute of Polymers, Bulgarian Academy of Sciences, Acad. G. Bonchev St, Bl. 103A, 1113 Sofia, Bulgaria; nstoyanova@polymer.bas.bg (N.S.); nachev_n@polymer.bas.bg (N.N.); 2 Centre of Competence “Sustainable Utilization of Bio-Resources and Waste of Medicinal and Aromatic Plants for Innovative Bioactive Products” (BIORESOURCES BG), 1000 Sofia, Bulgaria; 3 Institute of Experimental Morphology, Pathology and Anthropology with Museum, Bulgarian Academy of Sciences, Acad. G. Bonchev St, Bl. 25, 1113 Sofia, Bulgaria; georgieva_any@abv.bg (A.G.); reneta.toshkova@gmail.com (R.T.)

**Keywords:** electrospinning, quercetin, PLA, PEG, drug delivery, cancer treatment

## Abstract

**Background:** The plant extract of quercetin possesses valuable pharmacological properties. However, its high instability, poor water solubility, and low cell bioavailability has limited its medical applications. An innovative approach used to overcome these limitations is the QUE incorporation in suitable polymer carriers. **Methods:** In the present study, fibrous materials based on PLA or PLA/PEG loaded with the flavonoid quercetin (QUE) were obtained by the electrospinning technique. Diverse morphological, spectroscopic, physico-mechanical, and spectrophotometric methods were used to characterize the prepared electrospun mats. **Results:** The addition of hydrophilic PEG to the polymer matrix improved its wettability and assisted the more rapid release of QUE from the PLA/PEG fibrous mat than from the PLA one. The obtained fibrous mats possess good mechanical properties. Moreover, QUE-loaded electrospun mats exhibited high anticancer activity against HeLa cervical cancer cells, but lower toxicity to normal cells. **Conclusions:** The obtained perspective results revealed the potential of the obtained QUE-loaded materials to find applications for wound healing and cancer treatment.

## 1. Introduction

Bioactive substances, including polyphenols, tannins, flavonoids, alkaloids, vitamins, minerals, omega-3-fatty acids, bioactive proteins or peptides, probiotics, etc., have been extensively investigated for their possible uses in the pharmaceutical, nutraceutical, and functional food industries due to the growing demands for disease prevention and health improvement [1]. Bioactive compounds produced by plants are recognized as phytochemicals. They can be found in fruits, vegetables, grains, nuts, and herbs [2]. Numerous phytochemicals are recognized for their strong antioxidant effects [3,4], which help in preventing cell damage by neutralizing free radicals and reducing oxidative stress of low-density lipoproteins (the so-called bad cholesterol)—a key step in the development of atherosclerosis [5,6]. Apart from their well-documented antioxidant capabilities, they exhibit anti-inflammatory [7,8], antimicrobial [9,10], and anticancer properties [7,11], largely attributed to their enzyme inhibition [12,13,14] and interaction with receptors [15]. Their anticancer effects are related to the modulation of multiple processes involved in carcinogenesis, such as oxidative stress [16], inflammation [17], cell proliferation [18], apoptosis [19,20], and angiogenesis [21,22].

Quercetin, a flavonoid belonging to the polyphenol family, exemplifies all these benefits and stands out as a multifaceted compound with a plethora of therapeutic implications. It demonstrates remarkable anti-inflammatory actions by inhibiting cyclooxygenase [23] and lipoxygenase [24] enzymes, reducing prostaglandin [25] and leukotriene synthesis [24]. Beyond its anti-inflammatory activity, quercetin’s anticancer potential is a subject of intense research and promising results. Its anticancer properties are versatile, encompassing a range of mechanisms that target various stages of cancer development and progression. One of the primary mechanisms by which quercetin exerts its anticancer effects is through the induction of apoptosis [26], a programmed cell death process, in malignant cells. By triggering apoptosis, quercetin can eliminate cancer cells without harming surrounding healthy tissue—a selective action that is highly desirable in cancer treatment. By disrupting tumor angiogenesis, quercetin can starve cancer cells of the resources they need to grow and spread [27]. Moreover, its antioxidant properties play a crucial role in protecting cells from DNA damage caused by reactive oxygen species, which is a key factor in cancer development [28]. In biomedical contexts, quercetin’s ability to accelerate tissue regeneration [29] and protect against oxidative damage is particularly significant. Nevertheless, despite its many pharmacological benefits, quercetin’s application as a therapeutic molecule in clinical research is limited due to its high instability, poor water solubility and resulting low bioavailability [30]. An innovative approach to overcome these limitations is the use of both natural and synthetic polymers to encapsulate this active compound [31,32].

Many different methods have been used for encapsulating bioactive compounds such as spray drying, freeze drying, coacervation, co-crystallization, nanoemulsion, sol–gel, etc. [33]. Among various techniques, electrospinning has emerged as a promising method for fabricating nanofibers that can effectively encapsulate and protect sensitive molecules [34,35]. When encapsulated within electrospun fibers, quercetin can be released gradually, ensuring prolonged bioactivity and minimizing premature degradation [36]. In our previous study we show the successful incorporation of quercetin in cellulose acetate-based fibrous materials. The obtained electrospun quercetin-loaded fibers show high antioxidant activity and cytotoxic effects against cancer cells. However, the mechanical characteristics of the electrospun cellulose acetate-based fibrous mats were relatively small [37,38].

The combination of PLA and PEG in electrospun fibers offers a promising approach to enhance the bioactivity and functionality of quercetin-loaded materials, leveraging the complementary characteristics of these polymers to boost their overall therapeutic potential [39]. The hydrophobic nature of PLA provides a stable matrix with excellent physic-mechanical properties [40] for the encapsulation of quercetin, ensuring its protection and controlled release. On the other hand, the incorporation of hydrophilic PEG into the fiber matrix addresses two objectives at once: it improves the hydrophilicity of the fibers, which is crucial for cell adhesion and interaction in biomedical applications, and it also aids in the dispersion and solubilization of quercetin within the fiber structure. Furthermore, the tunable degradation rates of PLA and PEG allow for the design of fibers with tailored release kinetics, ensuring sustained delivery of quercetin over extended periods. This is particularly advantageous in cancer therapy, where prolonged exposure to therapeutic agents can enhance treatment efficacy while minimizing side effects [41,42,43]. Additionally, the mechanical properties of the PLA-PEG blend can be optimized to match the requirements of specific tissues, further broadening their applicability in regenerative medicine and wound healing. The synergistic effects of these polymers, combined with the antioxidant, anti-inflammatory, and anticancer properties of quercetin, have created a multifunctional platform with significant potential for addressing complex biomedical challenges. This sophisticated approach to drug delivery promises to advance the field of biomedical applications, providing an innovative and effective strategy for treating cancer and other diseases.

Therefore, the aim of the present work was to obtain quercetin-loaded PLA or PLA/PEG fibrous materials by electrospinning and to characterize the prepared materials by using morphological, spectroscopic, physico-mechanical, and spectrophotometric analysis. The cytotoxic effect against cancer and normal cells was studied as well in order to assess the potential of obtained materials for application in medicine.

## 2. Materials and Methods

### 2.1. Materials

In the present study, the following polymers were used: polylactic acid (PLA, Ingeo™ Biopolymer 4032D, NatureWorks, Minnetonka, MN, USA; Mw = 259,000 g mol^−1^, Mw/Mn = 1.94, as determined using size-exclusion chromatography with polystyrene standards) and polyethylene glycol (PEG 100,000, Serva, Heidelberg, Germany). Dichloromethane (DCM) and ethanol (abs. EtOH) used in the study were of analytical grade of purity and were supplied from Sigma-Aldrich (Darmstadt, Germany).

Dulbecco’s modified Eagle medium (DMEM) and fetal bovine serum (FBS) were purchased from Gibco-Invitrogen (Leicestershire, UK). Antibiotic solution (penicillin–streptomycin) was from Lonza (Verviers, Belgium). Dimethyl sulfoxide (DMSO), phosphate-buffered solution (PBS), and Trypsin–EDTA solution (2.5 g/L trypsin and 0.2 g/L EDTA) were obtained from AppliChem (Darmstadt, Germany); 3-[4,5-dimethylthiazol-2-yl]-2,3-diphenyl tetrazolium bromide (MTT) was from Sigma-Aldrich Chemie GmbH (Darmstadt, Germany); acridine orange (AO) and ethidium bromide (EtBr) were purchased from Merck (Darmstadt, Germany). All sterile plasticware was from Orange Scientific (Braine-l’Alleud, Belgium).

### 2.2. Cell Lines and Culture Conditions

The human cervical carcinoma HeLa (CCL-2) and mice embryo fibroblasts BALB/3T3 (CCL-163) were obtained from the American Type Culture Collection (ATCC, USA ATCC, Rockville, MD, USA). The cells were cultured in Dulbecco’s modified Eagle medium (DMEM), supplemented with 10% fetal bovine serum (FBS), 2 mM L-glutamine, penicillin 100 U/mL, and streptomycin 100 µg/mL in a humidified atmosphere of 95% air, 5% CO_2_, at 37 °C. The cells were grown as monolayers in 25 cm^2^ tissue culture flasks. After achieving 60–80% confluency, the cells were detached from the flasks with 0.25% trypsin-EDTA (dissolved in PBS, pH 7.4), counted, and resuspended in fresh media to the required concentration for each test.

### 2.3. Preparation of Fibrous Mats by Electrospinning

Four types of electrospun materials were obtained from PLA, PLA/QUE, PLA/PEG, and PLA/PEG/QUE solutions. The polymer concentration in all solutions was 10 wt%. The PLA/PEG ratio was 80/20 *w*/*w*. The ratio of PLA to PEG was selected by preliminary experiments [44]. QUE was 10 wt% to the polymer weight. For the dissolution of the polymers and QUE, a mixture of dichloromethane/ethanol 80/20 *v*/*v* was used. A 5 mL syringe fitted with a metal needle (size: 20-gauge) whose tip was connected to the positively charged electrode was then filled with the prepared solutions. A specifically designed high-voltage power source that could provide positive DC voltages between 10 and 30 kV was attached to the electrode. The fibers were deposited onto the grounded drum-collector (45 mm in diameter), rotating with speed 1000 rpm. The used distance between the tip of the needle and the drum was fixed at 15 cm. An infusion pump (NE-300 Just InfusionTM Syringe Pump, New Era Pump Systems Inc., Farmingdale, NY, USA) was used to ensure the constant feeding rate of 3 mL/h. The applied voltage was 25 kV [45]. All the electrospinning experiments were performed at a room temperature of 21 °C and a relative humidity of 52%.

### 2.4. Characterization

At room temperature (25 °C), the dynamic viscosity of the prepared spinning solutions was measured using a Brookfield DV-II+ Pro programmed viscometer fitted with a cone spindle for the one/plate option and a sample thermostatic cup.

The structure of the created fibrous mats was determined using scanning electron microscopy (SEM). All samples were vacuum-coated with gold using a Jeol JFC-1200 fine coater for 60 s prior to analysis using a Jeol JSM-5510 (JEOL Co., Ltd., Tokyo, Japan). Using ImageJ software, version 1.54g, at least 30 fibers from the SEM images were analyzed in order to calculate the mean fiber diameter and the diameter standard.

The materials’ IR spectra were recorded and ATR-FTIR spectroscopic measurements were performed using an IRAffinity-1 spectrophotometer (Shimadzu Co., Kyoto, Japan) equipped with a MIRacle™ ATR accessory (diamond crystal, penetration depth of the IR beam ~2 µm) from PIKE Technologies (Fitchburg, WI, USA). Infrared absorption spectra were recorded in the 600–4000 cm^−1^ region at a resolution of 4 cm^−1^. All of the spectra for H_2_O and CO_2_ were adjusted using the IRsolution program, version 1.04.

To study the surface wettability of the fiber materials, contact angle measurements were performed at 25 °C using an Easy Drop DSA20E (Krüss GmbH, Hamburg, Germany). The average contact angle was calculated using computer analysis after a sessile drop of deionized water (8 µL) was applied to the surface of fibrous samples (2 cm × 7 cm, cut in the collector rotation direction). Each sample was measured ten times.

A 50 N load cell and an INSTRON 3344 single-column mechanical testing equipment with Bluehill Universal software, version 3.11, were used to assess the mechanical characteristics of the fibrous samples. Tests were carried out using a strain rate of 10 mm/min and at 21 °C. The specimens under examination have dimensions of 20 mm in width, 60 mm in length, and ~350 µm in thickness. Using at least 10 specimens per sample, the linear section of the stress–strain curves was utilized to determine the average values of elongation at break (εB, %), tensile strength (σ, MPa), and Young’s modulus (E, MPa).

Quercetin content in the fibrous materials was determined by dissolving samples (1 cm^2^) in 10 mL of dichloromethane/ethanol (80/20 *v*/*v*). Then the absorbance at 373 nm was measured using a DU 800 spectrophotometer UV (Beckman Coulter, Brea, CA, USA). The QUE loading efficiency was calculated from the following equation:

Loading efficiency = (amount of loaded QUE/amount of QUE in the feed) × 100%

Acetate buffer with a pH of 5.5, a constant ionic strength of 0.1 (CH_3_COONa/CH_3_COOH), and Tween 80 (acetate buffer/Tween 80 = 99.2/0.8 *v*/*v*) was used to study the quercetin release profile in vitro at 37 °C. A shaking thermostatic water bath (Julabo SW23, Allentown, PA, USA) and stirring at 150 rpm was used to agitate the 100 mL buffer solution in which the tested mats were submerged at 150 rpm. At predetermined intervals, aliquots of the test solution were removed, and the absorbance of each was measured at a wavelength of 373 nm. A calibration curve (correlation coefficient R = 0.9995) for the mats in acetate buffer/Tween 80 (99.2/0.8 *v*/*v*), pH = 5.5, and constant ionic strength of 0.1, was used to determine the quantity of released quercetin.

### 2.5. MTT Cell Viability Test

MTT (3-[4,5-dimethylthiazol-2-yl]-2,5 diphenyl tetrazolium bromide) assay [46] was used to determine the effect of different fibrous samples (QUE incorporated in PLA and PLA/PEG mats) on cell viability (cervical carcinoma HeLa and mice embryo fibroblasts BALB/3T3). The MTT test is based on the conversion of tetrazolium salt into formazan crystals only by metabolically active cells; accordingly, this assay detects viable cells exclusively. Briefly, tumor and non-tumor cells were plated in a 96-well plate at a concentration of 1 × 10^4^ cells/100 µL/well and incubated overnight as previously stated (in Section 2.2) to form a monolayer. Then the medium was removed, and cells were cultivated in contact with fibrous samples for 24 and 72 h. Each variant was assayed by five measurements. Respective cells incubated only in medium and in the presence of QUE solution (100 μM/L) were used as controls. At the completion of the treatment, the cells were washed with PBS (pH 7.4), and 100 mL of MTT working solution (5 mg/mL MTT in PBS) was added to each well and the plates were further incubated at 37 °C for 3 h. Formazan crystals were dissolved by adding 100 μL/well of lysing ethanol/DMSO (1:1 *v*/*v*) solution. The absorbance (optical density) in control and sample-treated cells was measured spectrophotometrically at 580 nm using the ELISA microplate reader (TECAN, SunriseTM, Grödig/Salzburg, Austria). The cell viability was calculated as follows.

Cell viability (%) = OD (experimental)/OD (control) × 100

Data are presented as percentages of values detected in control cells cultured under the same conditions in the absence of test samples (negative controls). All MTT assays were performed in case of initial viability exceeding 99%, which was checked using trypan blue dye. All assays were performed in triplicate.

### 2.6. Fluorescent Microscopy Investigations

#### 2.6.1. Double Staining Assay with AO–EtBr

The cell morphology alterations were evaluated by acridine orange (AO) (3,6-dimethylaminoacridine) and ethidium bromide (EtBr) (3,8-diamino-5-ethyl-6-phenylphenanthridinium bromide) double-staining. AO penetrates normal and early apoptotic cells with intact membranes, fluorescing green. EtBr only enters cells with damaged membranes, such as late apoptotic and dead cells, emitting orange-red fluorescence. Dual AO/EB fluorescent staining allows for the distinction between normal cells, early and late apoptotic cells, and necrotic cells. Briefly, the cells were grown on sterile 13 mm-diameter cover glasses placed at the bottom in 24-well plates (2.0 × 10^5^ cells/well) for 24 h in CO_2_ incubator to form a cell monolayer. The next day, the tested samples and QUE solution at concentrations of 100 μM/L were added. Cells from the respective line, cultured only in medium, served as negative controls. After 24 h of incubation, the coverslips were removed and washed twice with phosphate buffered saline (PBS), and then were stained with fluorescent dyes AO (5 µg/mL) and EtBr (5 µg/mL) in PBS. Freshly stained cells were examined immediately under a fluorescence microscope (Leica DM 5000B, Wetzlar, Germany) within 10 min before the fluorescent color started to fade.

#### 2.6.2. DAPI Staining

Staining with DNA-binding dye 4′,6-Diamidine-2′-phenylindole dihydrochloride (DAPI) was employed to examine the nuclear morphology. The DAPI molecule can pass through an intact cytoplasmic membrane, making it a suitable agent for studying the nuclear morphology of both live and fixed cells. For this purpose, the cells were cultured on glass lamellae and treated with different formulations, as described in the previous paragraph. Upon completion of the incubation, the cells were fixed with methanol and incubated for 15 min at 37 °C with DAPI solution (1 µg/mL in methanol) in the dark. Stained cells on glass lamellae were coated with Mowiol^®^, mounted on slides, and observed under a fluorescence microscope (Leica DM 5000B, Wetzlar, Germany).

### 2.7. Statistical Analysis

The data were shown as means and standard deviations (SD). The GraphPAD PRISM software, version 5, was utilized to assess the statistical significance of the data using one-way analysis of variance (ANOVA) and the post hoc comparison test (Bonferroni) (GraphPad Software Inc., San Diego, CA, USA). Statistical significance was defined as * *p* < 0.05, ** *p* < 0.01, and *** *p* < 0.001.

## 3. Results and Discussion

Electrospinning is recognized as a technique capable of loading bioactive compounds/drugs within polymer fibrous materials in one step, which has attracted broad interest as an effective and efficient bioactive substance delivery system. Furthermore, it is proved that the use of this electrohydrodynamic process enhances the bioavailability of many water-insoluble bioactive compounds [35,47] because it results in the preparation of nano- and microfibers with high surface-to-volume ratio and high porosity [34].

### 3.1. Electrospinning of Fibrous Mats

In the present study, electrospinning of four different-in-composition spinning solutions was performed. Prior to electrospinning, the dynamic viscosities of the spinning solutions were measured. The values of the viscosities for the PLA, PLA/PEG, PLA/QUE, and PLA/PEG/QUE solutions were 1700 cP, 1245 cP, 1885 cP, and 1450 cP. As seen from the presented values, the dynamic viscosities depend on the presence of low-molecular-weight polymers, which in our case is PEG and the addition of the bioactive compound—quercetin. The addition of PEG to the PLA solution resulted in a dynamic viscosity decrease, while the incorporation of QUE to the PLLA or PLA/PEG solutions led to an increase in the viscosity values. Measuring the spinning solution viscosities is crucial in order to find the relationship composition/viscosity/fiber diameter. The viscosity of the solution is usually considered to be the main parameter which determines the fiber diameter. Generally, increasing concentration and, respectively, the solution viscosity results in larger fiber diameters.

**Figure 1:**
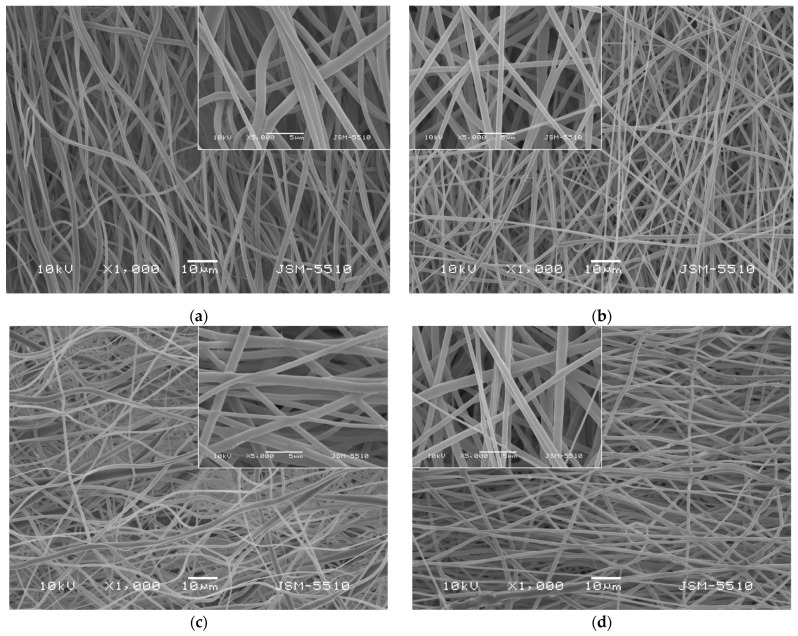
SEM micrographs of fibrous materials: (**a**) PLA, (**b**) PLA/PEG, (**c**) PLA/QUE, and (**d**) PLA/PEG/QUE.

The morphology of the prepared fibrous materials observed by using scanning electron microscopy (SEM) was shown in Figure 1. All the obtained fibers were defect-free and cylindrical. Using the captured SEM images, the average fiber diameters using the specialized software were determined. The electrospinning of PLA solutions with a concentration of 10 wt% in DCM/ethanol resulted in fabrication of PLA fibers with diameters of 768 ± 138 nm. The addition of a second much lower in molecular weight polymer PEG to the PLA solution reduced the dynamic viscosity of the blend solution as well as the resulting fiber diameters. The average value of the PLA/PEG fibers was determined to be 671 ± 137 nm. The quercetin loading into PLA and PLA/PEG fibers increased slightly the average fiber diameter compared to the neat PLA and PLA/PEG fibers to 807 ± 159 nm and 713 ± 143 nm, respectively.

### 3.2. ATR-FTIR Spectra of Fibrous Materials and QUE

**Figure 2:**
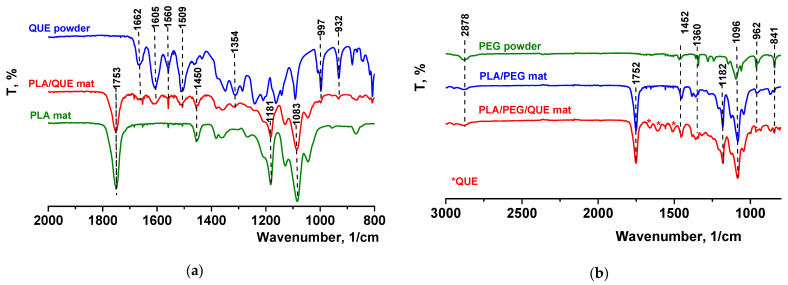
ATR-FTIR spectra of (**a**) QUE powder, PLA/QUE mat, and PLA mat and (**b**) PEG powder, PLA/PEG mat, and PLA/PEG/QUE mat. * QUE—peaks appearing from quercetin.

The FTIR spectrum for pure quercetin (powder) is shown in Figure 2a, where its characteristic bands were detected [48]. It revealed that the peak at 1605 cm^−1^ refers to stretching absorption from the C=O group, and 1240 cm^−1^ to C-H stretching. OH bending of the phenol function was detected at 1354 cm^−1^. The C=O aryl ketonic stretch absorption was evident at 1662 cm^−1^. C=C aromatic ring stretching bands were detectable at 1605, 1560, and 1509 cm^−1^. A weaker absorption in the 1314 cm^−1^ is due to in-plane C-H bending in aromatic hydrocarbon, and out-of-plane bending bands were evident at 932 and 600 cm^−1^. Bands between 1286 and 1163 cm^−1^ were attributable to the C-O stretching in the aryl ether ring, the C-O stretching in phenol, and the C-CO-C stretch and bending in ketone, respectively. The FTIR spectra of electrospun fibrous mats based on polylactic acid loaded with or without quercetin were also presented in Figure 2a. In the FTIR spectrum of the neat PLA mat, characteristic stretching frequencies for C=O, -CH_3_ asymmetric, -CH_3_ symmetric, and C-O at 1753, 2995, 2945, and 1084 cm^−1^ were presented [45]. In the spectrum of the PLA/QUE fibrous mat, characteristic bands for PLA (1753, 1450, 1181, and 1083 cm^−1^) as well as for the QUE (1662,1605, 1560,1509, 1354,997, and 932 cm^−1^) were detected. The presence of PEG in PLA/PEG and PLA/PEG/QUE mats resulted in detecting additional bands at 2878, 1452, 1360, and 962 cm^−1^, characteristic for PEG (Figure 2b). In the IR spectrum of PLA/PEG/QUE, fibrous material bands characteristic for PLA, PEG, and QUE are presented, proving the successful incorporation of the flavonoid in the PLA and PLA/PEG polymer matrixes.

### 3.3. Contact Angle Measurements and Wettability

One of the most simple and useful methods to characterize the wettability of the materials’ surface is the water contact angle measurements. The measured angle is the angle that a liquid forms at the intersection of a liquid, gas, and solid at the three-phase boundary point. In the present study, the static contact angle of fibrous substrates was determined by using a contact angle goniometer. The used method immediately shows the information about the wettability of the studied solid.

**Figure 3:**
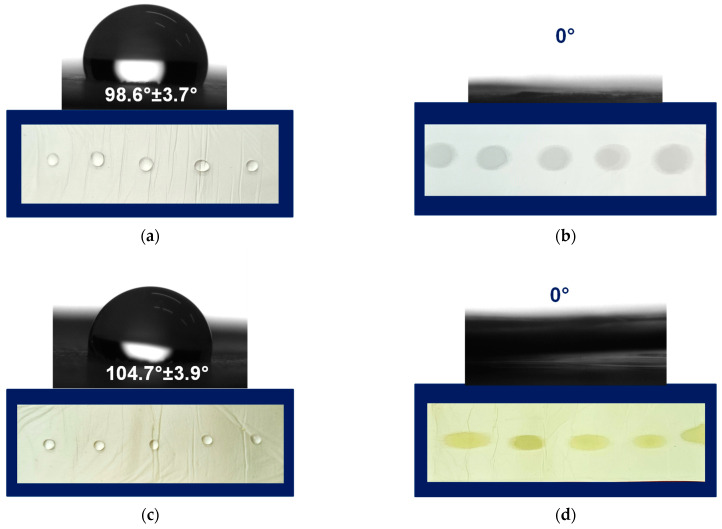
Images of water droplets deposited on the surface of (**a**) PLA, (**b**) PLA/PEG, (**c**) PLA/QUE, and (**d**) PLA/PEG/QUE.

Prior to the contact angle measures, the fibrous mats of PLA, PLA/PEG, PLA/QUE, and PLA/PEG/QUE were cut into a rectangular shape, and droplets with volume 7 μL were deposited onto the mats’ surface. Then the water droplets were captured, and the values of the water contact angles were measured. Figure 3 presents the captured water droplet shapes and the determined values. As could be easily seen, the mats based on PLA and PLA/QUE were hybrophobic with contact angles of 98.6° ± 3.7° and 104.7° ± 3.9° (Figure 3a,c). The incorporation of QUE into the PLA solution and after that into the fibers resulted in a slight increase in the water contact angle, which is probably due to its hydrophobic nature [30]. The hydrophobic nature of quercetin restricts its application as a bioactive compound and, therefore, novel approaches aiming to improve its solubility and bioavailability are sought. The incorporation of a water-soluble agent such as polyethylene glycol is a prospective way to tune the hydrophilic–hydrophobic balance of formulations containing natural polyphenols such as quercetin. This assumption was proved by the performed water contact angle measurements of the samples based on PLA/PEG and PLA/PEG/QUE. As presented in Figure 3b,d, the deposited water droplets immediately absorb into the fibrous material and the measured contact angle is 0°. Thus, the obtained PLA/PEG and PLA/PEG/QUE mats were superhybrophilic and wettable. This will assist the QUE release from the fibrous mats and will promote QUE to manifest its valuable biological properties.

### 3.4. Physico-Mechanical Testing

**Figure 4:**
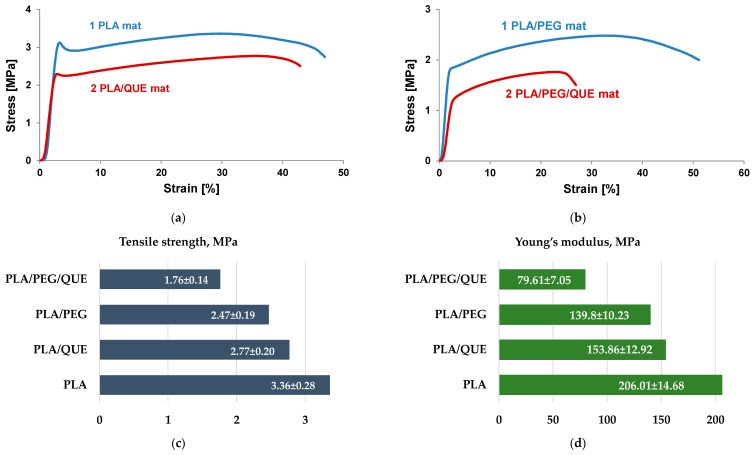
Mechanical characteristics of PLA, PLA/QUE, PLA/PEG, and PLA/PEG/QUE mats: (**a**) stress–strain curves of PLA and PLA/QUE mats, (**b**) stress–strain curves of PLA/PEG and PLA/PEG/QUE mats, (**c**) tensile strength, and (**d**) modulus of elasticity of all electrospun mats.

Mechanical characteristics of the materials play an important role in determining their applications. The mechanical properties of electrospun materials strongly depend on electrospinning conditions, fiber diameters, presence of defect/pores, orientation, bonding between fibers, crosslinking, etc. Furthermore, additional components such as bioactive molecules, nanoparticles, metal (nano)particles, and nanotubes influence the mechanical characteristics as well. Therefore, it is important to study the influence of a double polymer mixture and the addition of quercetin, which is a flavonoid with a low molecular weight. Mechanical characteristics of the electrospun PLA, PLA/QUE, PLA/PEG, and PLA/PEG/QUE mats are shown in Figure 4. The stress–strain curves of the PLA and PLA/QUE mat are presented in Figure 4a. PLA mats show the highest value of tensile strength of 3.36 MPa. The QUE loading in PLA fibrous mats resulted in some decrease in the tensile strength and Young’s modulus to 2.77 MPa and 153.86 MPa (Figure 4c,d). The observed decrease in the mechanical characteristics is due to the addition of a compound with low molecular weight. Furthermore, the addition of a second polymer with a much lower molecular weight of PLA resulted in a further decrease in the mechanical properties, as well. Regardless of the slight decrease in tensile strength and Young’s modulus, the overall mechanical characteristics of the PLA/QUE and PLA/PEG/QUE mats remain very good.

### 3.5. In Vitro Release Study

The flavonoid QUE has a low bioavailability, poor water solubility, and very little skin penetration ability. Micellization research has emerged as a promising solution to these challenges. Micellization is connected with aggregation formation in the presence of amphiphilic chemicals such as surfactants. Micelles can disguise quercetin’s poor characteristics by trapping its molecules within their micellar structure, increasing its solubility and permeability. Tween 80 is a non-ionic surfactant that is frequently utilized in the cosmetics and pharmaceutical industries. The non-ionic surfactant Tween 80 is used extensively in the pharmaceutical and cosmetics sectors due to its micelle-forming properties [49]. To evaluate the in vitro release profiles from QUE-loaded fibers, in vitro release tests were performed in the acetate buffer solution at a pH of 5.5 to mimic tumor environment pH [50].

**Figure 5:**
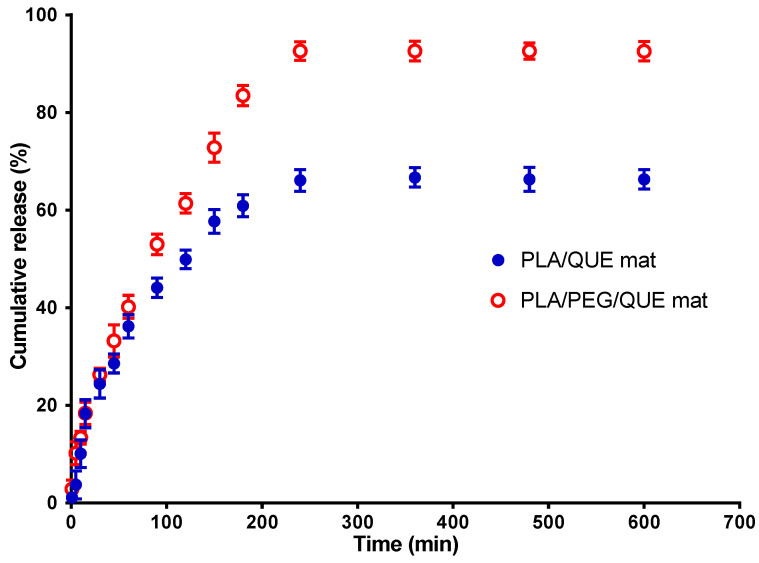
QUE release profile from PLA/QUE and PLA/PEG/QUE fibers. The results are presented as average values from three separate measurements with the respective standard deviation; acetate buffer/Tween 80 (99.2/0.8 *v*/*v*), pH 5.5, 37 °C, ionic strength 0.1.

The current investigation used a method for quercetin release in the presence of Tween 80 in order to simulate the release of QUE from the PLA/QUE and PLA/PEG/QUE fibrous mat. In the in vitro release study, we have used acetate buffer/Tween 80 = 99.2/0.8 *v*/*v*. Figure 5 reveals the QUE release profile. About 67% of QUE was released from the PLA/QUE fibers over 360 min, and this amount did not vary over the course of 24 h. For the PLA/PEG/QUE fibers, the amount of the released flavonoid was about 93% due to the composition of the polymer matrix. The release characteristics of encapsulated quercetin showed an early burst release, followed by a steady release throughout time. The release of QUE was considerably accelerated in PLA/PEG/QUE compared with that of the PLA/QUE (93% versus 67%). Incorporating a water-soluble polymer like PEG into biodegradable polyesters adds hydrophilicity to the polymer matrix and acts as a “water pump” [51], thereby enhancing the flavonoid’s release from the electrospun fibers [52,53]. Inclusion of PEG into the polymer matrix increases hydrophilicity and promotes water uptake, leading to facilitation of the escape of QUE molecules.

The results for the release of quercetin from PLA/QUE and PLA/PEG/QUE fibers were cross-checked by determining the encapsulation efficiency of QUE into the polymer fibers. For this purpose, the fibrous mats were dissolved in dichloromethane/ethanol and the absorbance of the obtained solution at 373 nm was recorded. It was found that the total amount of QUE in the PLA and PLA/PEG fibrous mats was 92% and 96%, respectively. These results indicate that QUE encapsulation efficiency was close to 100%.

### 3.6. In Vitro Cytotoxicity Tests

Data on anticancer potential of quercetin-loaded electrospun nanofibers are scarce. Most of the authors limited their studies to only one cancer cell line [54,55,56]. Therefore, more comprehensive studies are needed to prove that electrospun fibrous materials loaded with QUE may have the potential be used in cancer treatments. In our study, HeLa cancer cells and normal BALB/c 3T3 cells were tested. There are no studies in the literature concerning the anticancer activity of PLA or PLA/PEG electrospun fibers loaded with quercetin against HeLa cancer cells.

**Figure 6:**
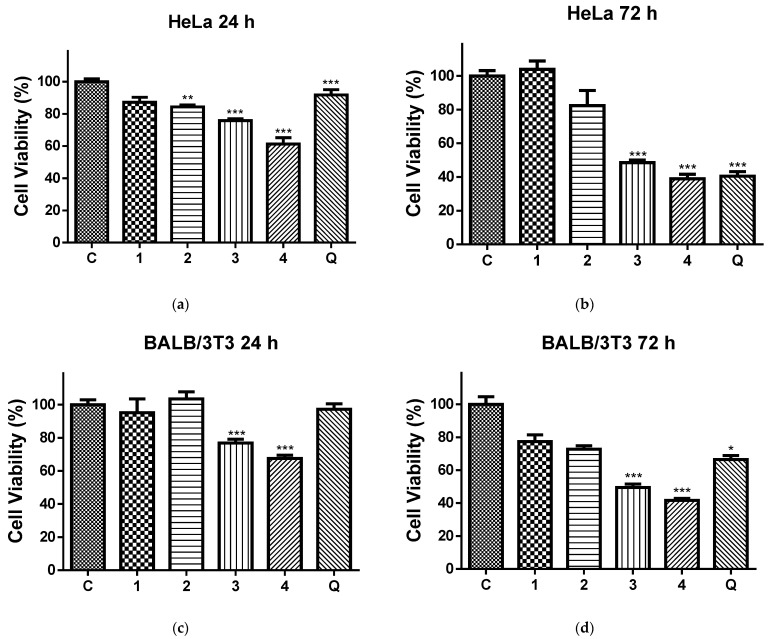
Cell viability of HeLa cells (**a**,**b**) and BALB/c 3T3 cells (**c**,**d**) after 24 h (**a**,**c**) and 72 h (**b**,**d**), after contact with C—control of untreated cells; 1—PLA mat; 2—PLA/PEG mat; 3—PLA/QUE mat; 4—PLA/PEG/QUE mat; and Q—free quercetin. * *p* < 0.05, ** *p* < 0.01, and *** *p* < 0.001.

Figure 6 shows the effects of the 24 h (Figure 6a,b) and 72 h (Figure 6c,d) contact of the HeLa (Figure 6a,c) and normal BALB/c 3T3 cells (Figure 6b,d) with the QUE solution and all four types of electrospun mats on the cell viability. After 24 h and 72 h of incubation with the PLA and PLA/PEG mat, the cell viabilities were 87.17% and 103.99% (for the PLA mats), 84.37% and 82.31% (for the PLA/PEG mats), respectively. Based on these results, we conclude that these mats have insignificant antiproliferative activity. In contrast, all QUE-containing mats exhibited strong anticancer properties against cervical cancer cells (HeLa) according to the MTT test data of in vitro cytotoxicity assays shown in Figure 6a,b. The QUE-loaded PLA and PLA/PEG mats showed a considerable antiproliferative impact. The percentage of viability of the HeLa cells was significantly decreased, in particular after 72 h of incubation. For that time period, the viability of HeLa cells was decreased to 48.53% and 38.97% for PLA/QUE and PLA/PEG/QUE mats, respectively. The highest antiproliferative activity compared to all other mats and the QUE solution was detected for the PLA/PEG/QUE mat. This is due to the fact that the obtained novel PLA/PEG/QUE mats contain the biological active compound in sufficient quantities (10 wt% to the polymer weight) in order to manifest its antitumor activity and moreover the incorporated PEG facilitates the solubility and the release of QUE. This finding corresponds with the results from the water contact angle, where the measured value for the PLA/PEG/QUE mat was 0°, and with the results for the release of quercetin (93%).

In addition, an MTT test with same time periods was performed using normal BALB/c 3T3 cells, as well. The results were presented in Figure 6c,d. This figure shows that normal cells were also affected upon contact with QUE-containing mats. However, the levels of toxicity against normal BALB/c 3T3 cells were lower compared to those in HeLa cells.

### 3.7. Assay of Cell Death by Staining Methods

**Figure 7:**
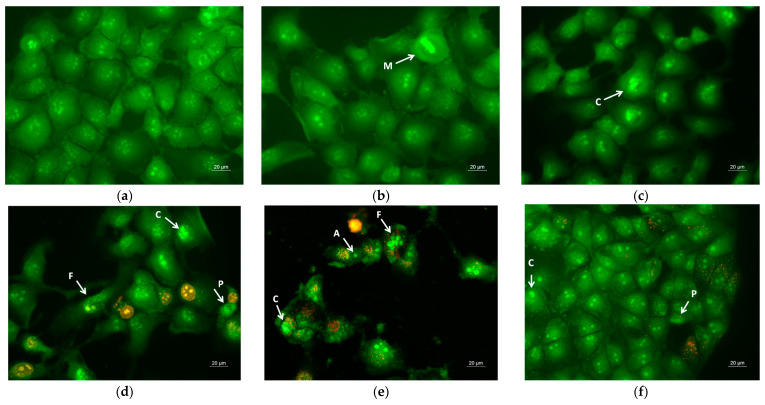
Images from fluorescence microscope of AO and EtBr double-stained HeLa cancer cells incubated for 24 h with (**a**) untreated cells, (**b**) PLA mats, (**c**) PLA/PEG mats, (**d**) PLA/QUE, (**e**) PLA/PEG/QUE mats, and (**f**) free QUE; A—apoptotic body; C—chromatin condensation; F—nuclear fragmentation; P—pyknosis; M—mitosis.

Apoptosis is a type of programmed cell death, and it is generally characterized by distinct morphological characteristics. Chromatin condensation, chromosomal DNA fragmentation into internucleosomal fragments, cell shrinkage, membrane blebbing, and the production of apoptotic bodies without plasma membrane collapse are the morphological characteristics of apoptosis [57]. Intravital double-labeling using fluorescent dyes (AO and EtBr) and DAPI staining were used to detect the morphological changes in damaged cells. Human cervical carcinoma HeLa were cultured for 24 h with the prepared PLA, PLA/PEG, PLA/QUE, and PLA/PEG/QUE mats and QUE solution and then were double-stained with AO and EtBr fluorescent dyes. The fluorescently stained cells are presented in Figure 7. HeLa cells that have not been treated (control) have a typical morphological structure, with vivid yellow-green nucleoli and light green nuclei. Normal morphology is detected for the HeLa cells in contact with PLA and PLA/PEG mats. The number of cells that contacted with the PLA/PEG mat were slightly reduced compared with those of the untreated cells and cells in contact with the PLA mat. This finding is most probably due to the fact that PEG possesses a cytostatic effect [58]; however, in Figure 7c, the cells have a normal morphological structure, and no dead cells were observed. The contact of HeLa cells with the QUE-containing mats resulted in cell morphological alternations. Cells’ nuclei and cytoplasms were stained in orange-red. Most of them had morphological features of either early (shrinkage, blebbing of plasma membrane) or late (chromatin condensation, DNA fragmentation, apoptotic bodies) apoptosis. Furthermore, the number of cells was significantly reduced compared with the number of HeLa control cells.

**Figure 8:**
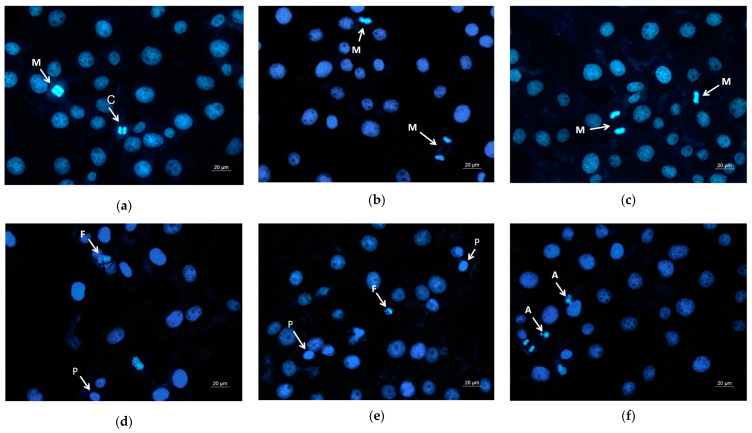
Images from fluorescence microscope of human cervical carcinoma HeLa stained with DAPI: (**a**) untreated cells, (**b**) PLA mats, (**c**) PLA/PEG mats, (**d**) PLA/QUE, (**e**) PLA/PEG/QUE mats, and (**f**) free QUE; A—apoptotic body; C—chromatin condensation; F—nuclear fragmentation; P—pyknosis; M—mitosis.

Moreover, DAPI staining of the HeLa cells that have been in contact with the mats or with the free QUE was performed as well. It is well known that DAPI is a fluorescent stain that binds strongly to adenine–thymine-rich regions in DNA and is used to observe alternations in nucleus of cells. Figure 8 presents the fluorescence microscopic images of the HeLa stained with DAPI. The nuclei of the untreated HeLa cells and those that were in contact with the PLA and PLA/PEG mats possessed smooth edges, and uniformly distributed and homogeneously colored chromatin. The cells that were in contact with QUE-containing mats and QUE solution displayed chromatin condensation, pyknosis of nuclei, and nuclei fragmentation. The cells with altered nuclei were found to be greatest in the cells in contact with the PLA/PEG/QUE mat. The observed results from the staining assay are in good agreement with the results obtained from the MTT test. These findings indicate that the QUE-containing mats cause cell death through the apoptosis pathway. The results suggest that the obtained fibrous PLA and PLA/PEG mats loaded with QUE are promising candidates for local drug delivery in cervical cancer treatment.

**Figure 9:**
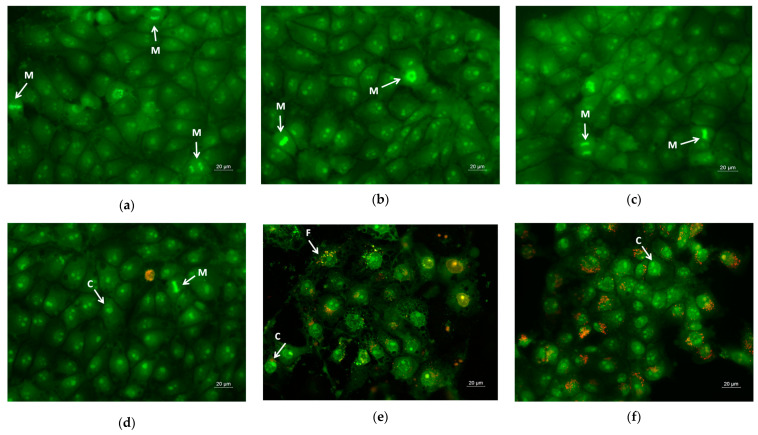
Images from fluorescence microscope of AO and EtBr double-stained mice embryo fibroblasts BALB/3T3 incubated for 24 h with (**a**) untreated cells, (**b**) PLA mats, (**c**) PLA/PEG mats, (**d**) PLA/QUE, (**e**) PLA/PEG/QUE mats, and (**f**) free QUE; C—chromatin condensation; F—nuclear fragmentation; M—mitosis.

Furthermore, for the sake of comparison, normal cells were also placed in contact with those prepared in the study’s fibrous mats. The study of the cytotoxicity of the obtained materials toward the normal cells was performed using mice embryo fibroblasts BALB/3T3. Figure 9 presented the fluorescent images from the double staining of the normal cells after being in contact with PLA, PLA/PEG, PLA/QUE, and PLA/PEG/QUE mats and free QUE. Untreated fibroblasts and those cultured with PLA and PLA/PEG mats possessed normal morphology and were green-stained. The monolayer of normal cells contacted with the neat polymer mats (Figure 9b,c) was similar to that of the control cells (Figure 9a). This is an indication that the polymers themselves do not affect the cell growth and morphology. Some moderate alternations were observed after contact with PLA/QUE and PLA/PEG/QUE mat cells with signs of early apoptosis, and single ones with signs of late apoptosis were observed. However, the fibroblast number was not significantly affected, nor was the number of HeLa cells after contact with PLA/QUE and PLA/PEG/QUE mats. The cells in contact with quercetin solution showed disorder of the nuclear chromatin characteristic of apoptosis.

The obtained results from the MTT cell viability test, double-staining assay with AO–EtBr, and DAPI staining reveal that PLA/QUE, and especially PLA/PEG/QUE fibrous mats, caused cell death by apoptosis. They showed higher toxicity to HeLa cancer cells and lower to normal fibroblast cells. These features make the prepared materials prospective candidates for application in medicine as wound dressings and in the local treatment of tumors. Moreover, the obtained results from MTT and staining methods are similar to those presented by Hudecki et. al. In their work, the authors study the anticancer action of polylactic acid-based materials containing quercetin using two types of cancer and nine cell lines, namely osteosarcoma (MG-63, U-2 OS, SaOS-2 cells) and breast cancer (SK-BR-3, MCF-7, MDA-MB-231, MDAMB-468, Hs 578T, and BT-20 cells). The anticancer activity of quercetin-loaded fibers was more pronounced than the action of free quercetin. PLA-based fibers promoted cell cycle arrest, oxidative stress, and apoptotic cell death that were not overcome by the heat shock protein (HSP)-mediated adaptive response. The obtained fibers were biocompatible and safe, as judged by in vitro testing using normal fibroblasts [56].

## 4. Conclusions

PLA and PLA/PEG fibrous materials loaded with QUE were obtained by the electrospinning technique. Inclusion of PEG into the polymer matrix increases hydrophilicity and promotes water uptake, leading to facilitation of the escape of QUE molecules. In addition, the loaded bioactive compound QUE imparted valuable anticancer activity to the fibrous mats. While maintaining lower toxicity against normal mouse BALB/c 3T3 fibroblasts, the QUE-loaded PLA and PLA/PEG mats had strong antiproliferative action against human cervical HeLa carcinoma cells. The obtained favorable results reveal the potential of the prepared novel materials to be used in wound healing and cancer treatment applications. Therefore, further research focused on structural analysis, antioxidant activity, and antibacterial activity of the obtained materials is planned to be performed.

## Data Availability

The data are contained within this article.
